# *Caenorhabditis elegans dnj-14*, the orthologue of the *DNAJC5* gene mutated in adult onset neuronal ceroid lipofuscinosis, provides a new platform for neuroprotective drug screening and identifies a SIR-2.1-independent action of resveratrol

**DOI:** 10.1093/hmg/ddu316

**Published:** 2014-06-19

**Authors:** Sudhanva S. Kashyap, James R. Johnson, Hannah V. McCue, Xi Chen, Matthew J. Edmonds, Mimieveshiofuo Ayala, Margaret E. Graham, Robert C. Jenn, Jeff W. Barclay, Robert D. Burgoyne, Alan Morgan

**Affiliations:** Department of Cellular and Molecular Physiology, Institute of Translational Medicine, University of Liverpool, Crown St, Liverpool L69 3BX, UK

## Abstract

Adult onset neuronal lipofuscinosis (ANCL) is a human neurodegenerative disorder characterized by progressive neuronal dysfunction and premature death. Recently, the mutations that cause ANCL were mapped to the *DNAJC5* gene, which encodes cysteine string protein alpha. We show here that mutating *dnj-14*, the *Caenorhabditis elegans* orthologue of *DNAJC5*, results in shortened lifespan and a small impairment of locomotion and neurotransmission. Mutant *dnj-14* worms also exhibited age-dependent neurodegeneration of sensory neurons, which was preceded by severe progressive chemosensory defects. A focussed chemical screen revealed that resveratrol could ameliorate *dnj-14* mutant phenotypes, an effect mimicked by the cAMP phosphodiesterase inhibitor, rolipram. In contrast to other worm neurodegeneration models, activation of the Sirtuin, SIR-2.1, was not required, as *sir-2.1*; *dnj-14* double mutants showed full lifespan rescue by resveratrol. The Sirtuin-independent neuroprotective action of resveratrol revealed here suggests potential therapeutic applications for ANCL and possibly other human neurodegenerative diseases.

## INTRODUCTION

Age-related neurodegenerative diseases have a devastating impact on affected individuals and pose a significant economic challenge for society. There is a pressing need to identify molecular and cellular neuroprotective mechanisms that decline with ageing so that drugs can be developed to modulate the neurodegenerative process and hence extend health-span. Human genetic studies have successfully identified single gene mutations in various neurodegenerative disorders. For example, frontotemporal dementia, Parkinson's and Huntington's diseases are associated with alterations in genes encoding tau, α-synuclein and huntingtin, respectively (1). Because misfolding and aggregation of these and other proteins is associated with disease progression, it is thought that defective protein homeostasis is a common underlying feature of neurodegeneration (2). Indeed, studies on animal models using genetic or pharmacological approaches have shown that altering the activity of molecular chaperones, proteasomes and autophagy can be neuroprotective (2–5). Drugs targeting these processes therefore represent promising candidate therapies.

Adult onset neuronal lipofuscinosis (ANCL), also known as autosomal dominant Kufs disease and Parry disease, is a hereditary neurodegenerative disorder (6). There is broad clinical variability, but common signs include generalized epilepsy, movement disorders and progressive dementia. The disease has a mean age of onset of 30 years and progresses rapidly, with death occurring on average at 45 years of age (7). Pathologically, ANCL is associated with intra-neuronal inclusions and neurodegeneration. Recently, several independent groups have discovered that ANCL is caused by mutations in the *DNAJC5* gene (7–10).

*DNAJC5* encodes an evolutionarily conserved member of the DnaJ/Hsp40 family of molecular chaperones known as cysteine string protein (CSP) (11,12). Its name derives from the possession of a motif containing 12–14 cysteine residues, palmitoylation of which is required for targeting of CSP to synaptic vesicles (13). ANCL patients all harbour mutations in *DNAJC5* that cause substitution or deletion of a pair of leucine residues within this cysteine string motif. These are effectively functionally null alleles, as the mutations have been shown to greatly reduce synaptic CSP levels and to phenocopy other *DNAJC5* loss-of-function mutants (8,14,15). Mouse *dnajc5* null mutants are characterized by age-dependent sensorimotor dysfunction, neurodegeneration and premature mortality, and exhibit synthetic genetic interactions with α-synuclein (16,17). It is thought that CSP acts as a specialized synaptic molecular chaperone, maintaining SNAP-25 in a conformation competent for entry into the fusogenic SNARE complex (18–20), although re-folding of other client proteins such as dynamin 1 is also likely to play a role (21). In *DNAJC5* mutants, the loss of this synaptic chaperone activity is thought to lead to neurodegeneration.

*Caenorhabditis elegans* has been used to model various neurodegenerative diseases, mainly by overexpressing human genes containing disease-associated mutations (22,23). Although some common genetic modifiers have been identified among these models, many of the underlying cellular mechanisms affected appear to be disease-specific, despite the shared feature of protein misfolding/aggregation (3,24). In order to identify generally neuroprotective interventions, an alternative approach is to search for compounds that are able to compensate for the loss of an endogenous physiological neuroprotective factor (25). Here we describe such a model, using *C. elegans* containing loss of function mutations in *dnj-14* (the worm orthologue of the *DNAJC5* gene mutated in ANCL); and demonstrate its potential for chemical genetic screening, by identifying a *sir-2.1*-independent neuroprotective effect of resveratrol.

## RESULTS

### Phenotypic consequences of impaired *dnj-14* function

The *C. elegans* genome encodes a single homologue of the *DNAJC5* gene that is mutated in ANCL: *dnj-14*. A comparison of the human and worm predicted proteins reveals extensive sequence similarity in the J domain and central cysteine string region, with more divergence evident in the C-termini (Supplementary Material, Fig. S1A). To determine if this structural similarity extends to function, we characterized a mutant strain (*ok237* allele), which contains a 2229 bp deletion that eliminates the majority of the *dnj-14* (KO2G10.8) coding region and its putative promoter (Supplementary Material, Fig. S1B and C). *dnj-14*(*ok237*) worms were viable and fertile, although they produced less progeny and development from egg to adult was slower than wild-type N2 control worms. Once developed into young adults, *dnj-14*(*ok237*) worms were superficially normal, as evidenced by their locomotion behaviour on agar plates (Supplementary Material, Movie S1).

**Figure 1.**
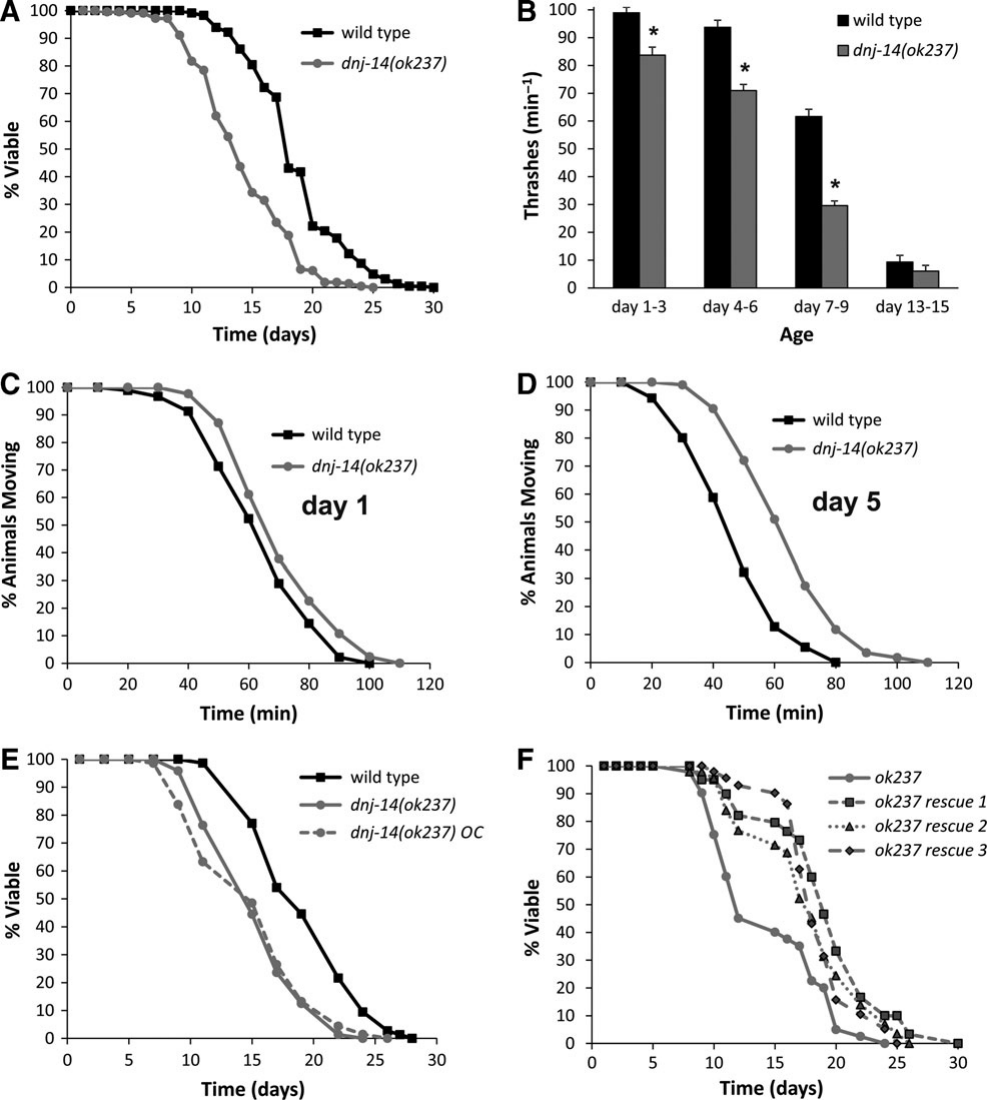
Phenotypic analysis of *dnj-14*(*ok237*) mutants. (**A**) *dnj-14* mutants have a short lifespan. Data shown are pooled from seven independent lifespan assays comparing synchronized wild-type (Bristol N2) and *dnj-14*(*ok237*) strains (*n* = 230 and 213 worms, respectively). (**B**) *dnj-14* mutants have a minor locomotion defect. The rate of thrashing in solution was measured in animals of the indicated age ranges after synchronization. Data are shown as mean ± SEM from two independent assays (*n* = 20–50 worms of each strain per time point; *P < 0.05). (**C** and **D**) *dnj-14* mutants have a minor, age-dependent neurotransmission defect. The time taken for paralysis to be induced by aldicarb was not significantly different from N2 controls in 1-day-old *dnj-14* mutants (C), but was significantly delayed in 5-day-old (D) animals. Data shown are pooled from 3 independent assays (*n* = 60 worms for each strain). (**E**) Out-crossed *dnj-14* mutants retain a short lifespan. The *dnj-14*(*ok237)* strain was out-crossed six times with the wild-type N2 strain and then lifespan analysis performed. Data shown are pooled from three independent lifespan assays comparing synchronized out-crossed (*OC*) and non-out-crossed strains (*n* = 68–74 worms for each strain). (**F**) The short lifespan of the *dnj-14* strain can be rescued by a *dnj-14* transgene. A construct containing the coding region and putative upstream promoter of the *dnj-14 gene* was injected into *dnj-14*(*ok237*) worms and then lifespan analysis performed. Data shown are from a single lifespan assay (*n* = 40, 32, 34 and 26 worms for untransformed, rescue 1, rescue 2 and rescue 3 strains, respectively).

As mutations in *DNAJC5* homologues are associated with reduced lifespan in humans (7–10), mice (16) and flies (11), we performed lifespan analysis on *dnj-14*(*ok237*) and wild-type N2 worms. In each of seven independent experiments performed by multiple individuals, *dnj-14*(*ok237*) mutants exhibited shorter lifespan than wild-type N2 worms. Similar results were seen whether FUDR (to prevent progeny development) was included in the NGM plates or omitted. Over this series, the mean lifespan of the *dnj-14*(*ok237*) strain was 13.3 days [95% confidence interval (CI): 12.8–13.8] compared with 18.7 days (95% CI: 18.2–19.2) for the wild-type N2 strain (Fig. 1A). We therefore conclude that deletion of *dnj-14* causes a reproducible and significant lifespan reduction.

Although *dnj-14*(*ok237*) worms exhibited superficially normal movement on agar plates (Supplementary Material, Movie S1), quantification of locomotion in solution revealed a small, but significant, reduction in thrashing activity (Fig. 1B) that became more pronounced with ageing. To determine if the impairment of locomotion was associated with a reduction in neurotransmitter release, we assayed the time taken for paralysis to be induced by the acetylcholinesterase inhibitor, aldicarb using established procedures (26). There was no significant difference in aldicarb assays between wild-type and mutant 1-day-old worms (Fig. 1C), but older *dnj-14* mutants displayed a significant delay in the time taken to become paralyzed (Fig. 1D). This rightward shift in the curve is indicative of a Ric (resistance to inhibitors of cholinesterase) phenotype, suggesting that *dnj-14* mutants exhibit a progressive, minor impairment of neurotransmitter release. The reduced lifespan of the *ok237* strain persisted despite back-crossing six times with the N2 control strain (Fig. 1E) and could be significantly increased by transgenic expression of *dnj-14* under the control of its putative natural promoter (Fig. 1F).

**Figure 2.**
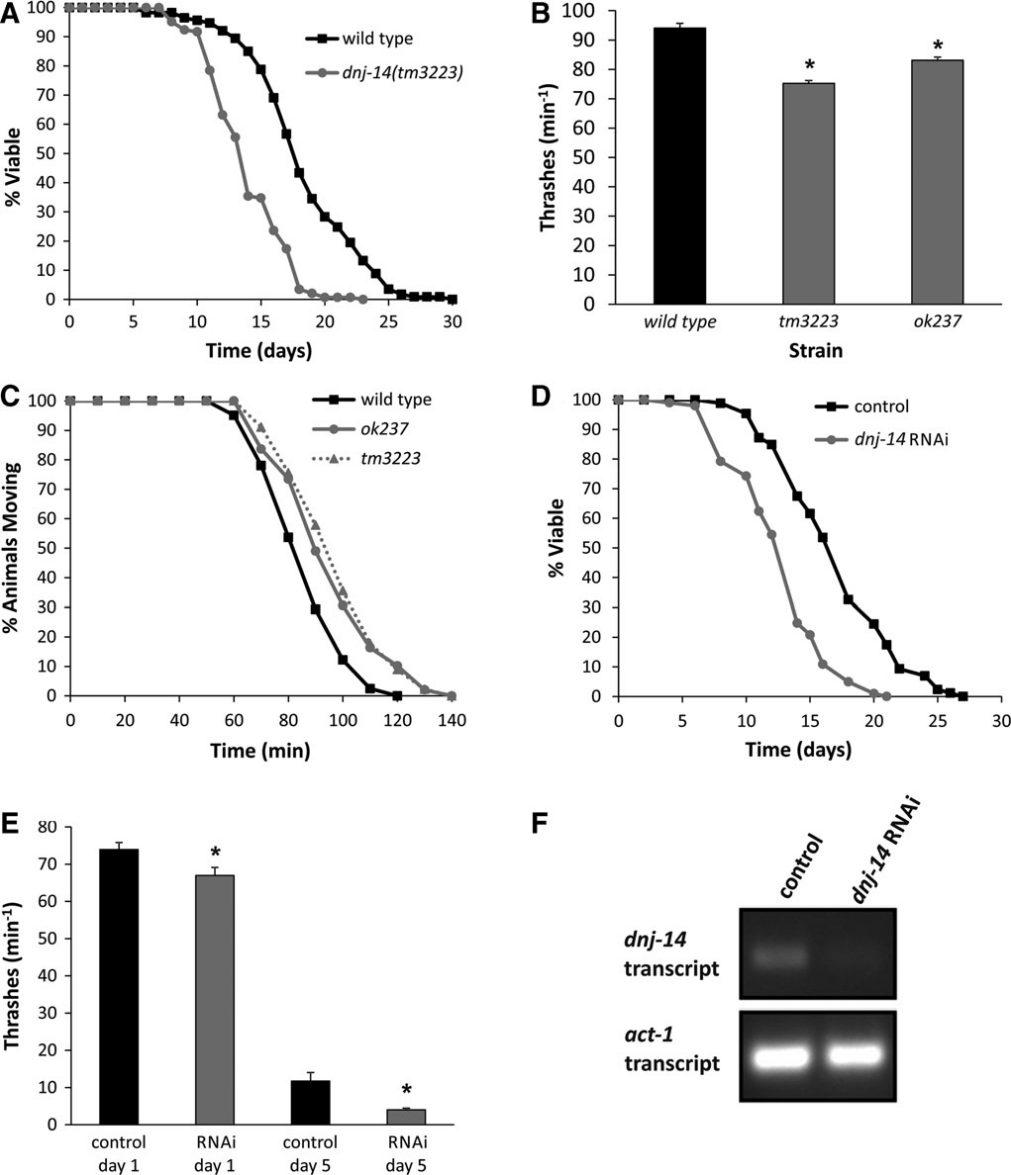
Multiple *dnj-14* alleles and RNAi produce similar phenotypes. (**A**) *dnj-14*(*tm3223*) mutants have a short lifespan. Data shown are pooled from four independent lifespan assays comparing synchronized wild-type (N2) and *dnj-14*(*tm3223*) strains (*n* = 113 and 144 worms, respectively). (**B**) Distinct *dnj-14* alleles produce a similar small locomotion defect. The rate of thrashing in solution was measured in 5-day-old animals. Data are shown as mean ± SEM from two independent assays (*n* = 35 worms for each strain; *P < 0.05). (**C**) Distinct *dnj-14* alleles produce a similar small neurotransmission defect. The time taken for paralysis to be induced by aldicarb was measured in 5-day-old animals. Data shown are pooled from two independent assays (*n* = 41, 49 and 45 worms for N2, *ok237* and *tm3223* strains, respectively). (**D**) RNAi of *dnj-14* shortens lifespan. RNAi-sensitive *eri-1* strains were maintained on control NGM plates or plates containing bacteria harbouring a *dnj-14* RNAi feeding plasmid. Data shown are pooled from two independent lifespan assays (*n* = 86 and 101 worms for control and *dnj-14* RNAi, respectively). (**E**) RNAi of *dnj-14* causes a small locomotion defect. The rate of thrashing in solution of *eri-1* worms was measured in control or RNAi-treated animals of the indicated ages. Data are shown as mean ± SEM from individual assays (*n* = 15 worms of each strain per age-point; *P < 0.05). (**F**) Validation of RNAi knockdown. RNA was prepared from *eri-1* worms (control and RNAi-treated) and used to generate cDNA for subsequent RT-PCR amplification using primers specific for *dnj-14* and *act-1*.

In addition to deleting *dnj-14*, the *ok237* allele also extends into the adjacent *glit-1* gene (Supplementary Material, Fig. S1B), raising the possibility that the observed phenotypes may be due to effects on *glit-1* rather than *dnj-14*. To rule this out, we obtained a second allele, *tm3223*, where the mutation was confined to *dnj-14* alone. Genomic DNA from the *dnj-14*(*tm3223*) strain was prepared and the *dnj-14* gene was PCR amplified and sequenced, revealing a 233 bp deletion and an insertion of five adenines within exon 2 of *dnj-14*. This would be predicted to produce a truncated protein terminating immediately after the J domain of DNJ-14. Over a series of 4 independent experiments performed by multiple individuals, *dnj-14*(*tm3223*) mutants consistently exhibited shorter lifespan than wild-type N2 worms, the mean lifespan of the *tm3223* strain being 14.0 days (95% CI: 13.46–14.44) compared with 18.3 days (95% CI: 17.5–19.1) for the N2 strain (Fig. 2A). We also found that *dnj-14*(*tm3223*) mutants displayed reduced locomotion in thrashing assays (Fig. 2B) and impaired neurotransmission in aldicarb assays (Fig. 2C), thus phenocopying the *dnj-14*(*ok237*) strain. To further confirm that these phenotypes were due to impairment of DNJ-14 function, we knocked down *dnj-14* expression using the RNAi-sensitive *eri-1* strain (27). This resulted in a lifespan reduction of similar magnitude to that seen with the *ok237* and *tm3223* alleles (Fig. 2D). Knockdown of *dnj-14* also recapitulated the small decrease in thrashing observed in the mutant strains (Fig. 2E). Although the control *eri-1* strain showed a much more severe reduction in locomotion during ageing than the N2 control strain, *dnj-14* RNAi exacerbated this further (Fig. 2E), consistent with results from the mutant strains. Confirmation that the RNAi vector used did indeed reduce the levels of *dnj-14* gene expression was provided via reverse transcriptase-polymerase chain reaction (RT-PCR) (Fig. 2F).

### Sensory neurons are impaired in *dnj-14* mutants

**Figure 3.**
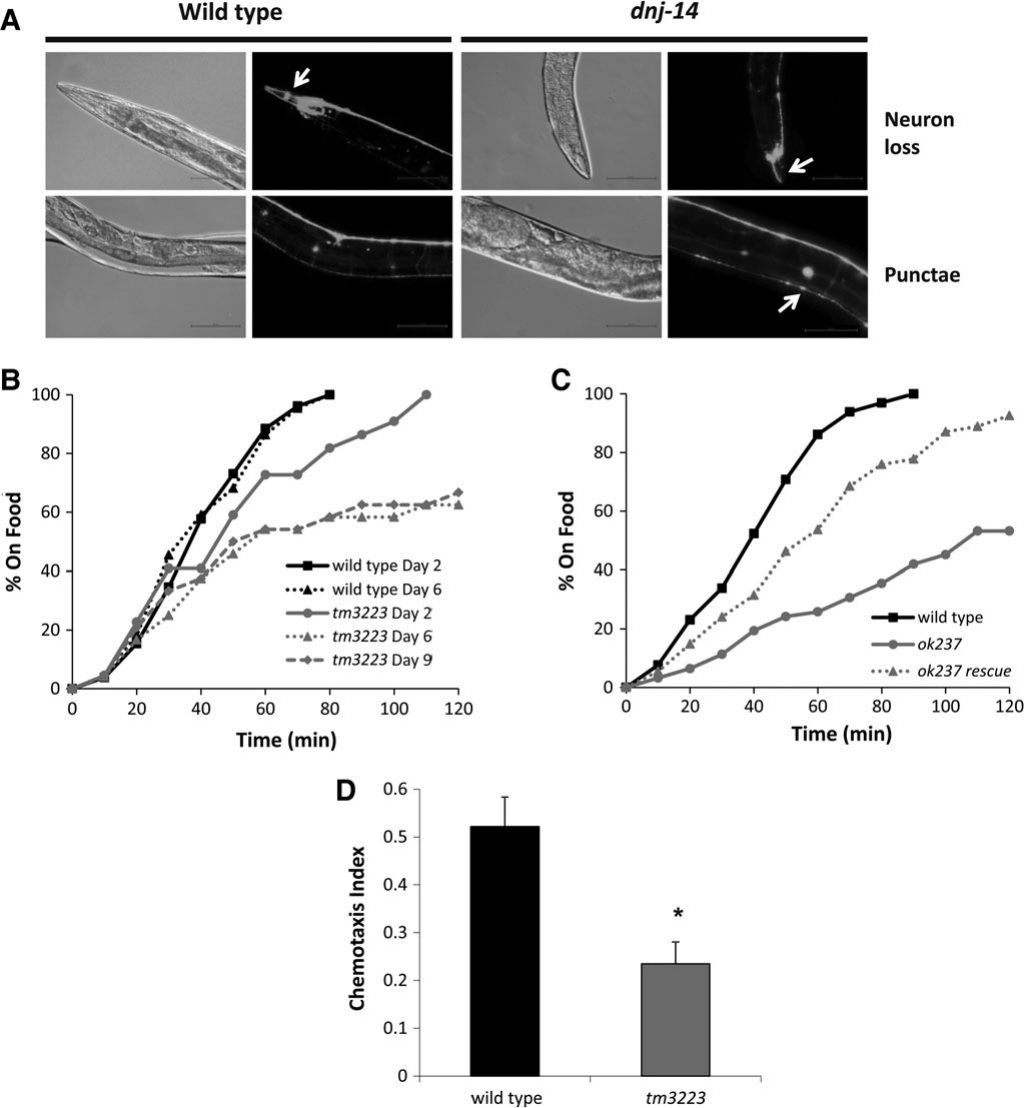
Age-dependent sensory neuron impairment in *dnj-14* mutants. (**A**) Neurodegeneration in *dnj-14*(*ok237*) mutants. Worms were injected with a *rab-3*-promoter-driven GFP construct to visualize all neurons, synchronized and grown for at least 9 days and then immobilized for GFP imaging. Representative images of the loss of cell bodies and processes in the head region (*neuron loss*) and the presence of large fluorescent aggregates in the dorsal nerve cord (*punctae*) that were typically observed are shown. Scale bar 50 µm. (**B**) Food sensing defect in *dnj-14* mutants. The time taken for wild-type and *dnj-14*(*tm3223*) worms of the indicated age to move to a bacterial food source was measured. Data shown are from individual assays, using 22–26 worms of each strain per age-point. (**C**) Transgenic rescue of the *dnj-14* food sensing defect. The time taken to move to a bacterial food source was measured in 6-day-old wild-type, *dnj-14*(*ok237*) and *dnj-14*(*ok237*) rescue strains (the latter containing an extrachromosomal *dnj-14* transgene). Data shown are pooled from two independent assays (*n* = 54–65 worms per strain). (**D**) Chemotaxis defect in *dnj-14* mutants. The proportion of wild-type and *dnj-14*(*tm3223*) worms that had moved to the attractant, isoamyl alcohol, within 90 min was measured in 2-day-old animals. Data shown are pooled from six independent assays (*n* = 168–170 worms of each strain; *P < 0.05).

Having established that the phenotype of the *ok237* strain is due to loss of *dnj-14* function, we used this null mutant strain to investigate effects on neurodegeneration, which is a feature of ANCL in humans. We employed the well-established approach of using free cytoplasmic green fluorescent protein (GFP) expressed from a neuron-specific promoter as a marker for neuronal viability and morphology, similar to previous studies (28,29). Live neurons were visualized in wild-type and *dnj-14*(*ok237*) animals by gonadal microinjection of a plasmid encoding GFP driven by the pan-neuronal *rab-3* promoter. As can be seen in Figure 3A, this results in labelling of all neuronal cell bodies and neurites in the worm, with neurons in the head, the nerve ring and the nerve cords being most apparent. There was no discernible difference in GFP labelling between wild-type and *dnj-14*(*ok237*) worms at <7 days of age. However, we observed notable neuronal abnormalities in the anterior head region of older *dnj-14* animals (Fig. 3A and Supplementary Material, Fig. S3). This manifested as a loss of neuronal cell bodies, a reduction in the number of visible neurites or the presence of contorted neuronal processes in the head of the worms. In contrast, age-matched wild-type N2 animals generally exhibited obvious neuronal cell bodies and had clearly labelled multiple neurites that extended straight to the end of the worm's head without twisting. We also observed an increase in the proportion of *dnj-14* animals with large GFP punctae in the dorsal nerve cord (Fig. 3A and Supplementary Material, Fig. S3); however, this phenotype was more variable and the difference between wild-type and mutant worms less obvious for than for the head neuron abnormalities described above. The nature of these punctae is not clear, although given that lysosomal inclusions are a feature of ANCL and that GFP fluorescence is resistant to lysosomal degradation (30), it is conceivable that they reflect accumulations of GFP within lysosomes. To quantify these phenotypes, worms from 9 days of adulthood and older were imaged and scored for head neuron abnormalities (‘neuron loss’) and the presence of dorsal nerve cord GFP aggregates (‘punctae’) (Supplementary Material, Fig. S2). This revealed that ∼70% of *dnj-14* mutants exhibited alterations in head neuron staining compared with only 9% of wild-type worms, representing an ∼8-fold increase. GFP punctae were evident in ∼30% of aged control animals, with this proportion increasing to ∼60% of *dnj-14* mutants. We therefore conclude that loss of *dnj-14* function results in age-dependent neurodegeneration that primarily affects anterior head neurons.

Given that the *C. elegans* head is rich in sensory neurons, we reasoned that this could affect the animal's ability to sense and react to environmental cues. Mechanosensation, as assayed by touch to both the nose and the side of the head, was no different between wild-type, *dnj-14*(*tm3223*) and *dnj-14*(*ok237*) strains at 6-days old (Supplementary Material, Fig. S4). Pharyngeal pumping was similarly unaffected in either allele at 6-days of age (Supplementary Material, Fig. S5), indicating that dnj-14 mutants are neither generally sick nor defective in functions of the worm head region. In contrast, *dnj-14*(*tm3223*) worms were severely impaired in a food race assay, which determines the time taken for animals to move a defined distance to a bacterial food source (Fig. 3B). This defect was already evident in young (Day 2) animals and was progressive, such that most day-6 *dnj-14*(*tm3223*) mutants appeared unaware of the location of the food source, in contrast to age-matched N2 controls. The same phenotype was seen with the *dnj-14*(*ok237*) allele and this could be rescued to close to control levels by transgenic expression of *dnj-14* (Fig. 3C). The observed defect in the food race assay was too severe to be explained by the minor locomotor defect in the animals, suggesting that a specific effect on chemosensation/olfaction was involved. To test this directly, chemotaxis assays using the well-established attractant, isoamyl alcohol, were employed (Fig. 3D). These revealed that *dnj-14*(*tm3223*) mutants have a severe deficiency in detecting and reacting to this volatile attractive compound. Given that profound defects in food race and chemotaxis assays were evident in young animals at ages where neuronal GFP staining was normal, this suggests that functional impairment precedes neurodegeneration in *dnj-14* mutants.

### Screen for compounds that rescue *dnj-14* phenotypes identifies resveratrol

**Figure 4.**
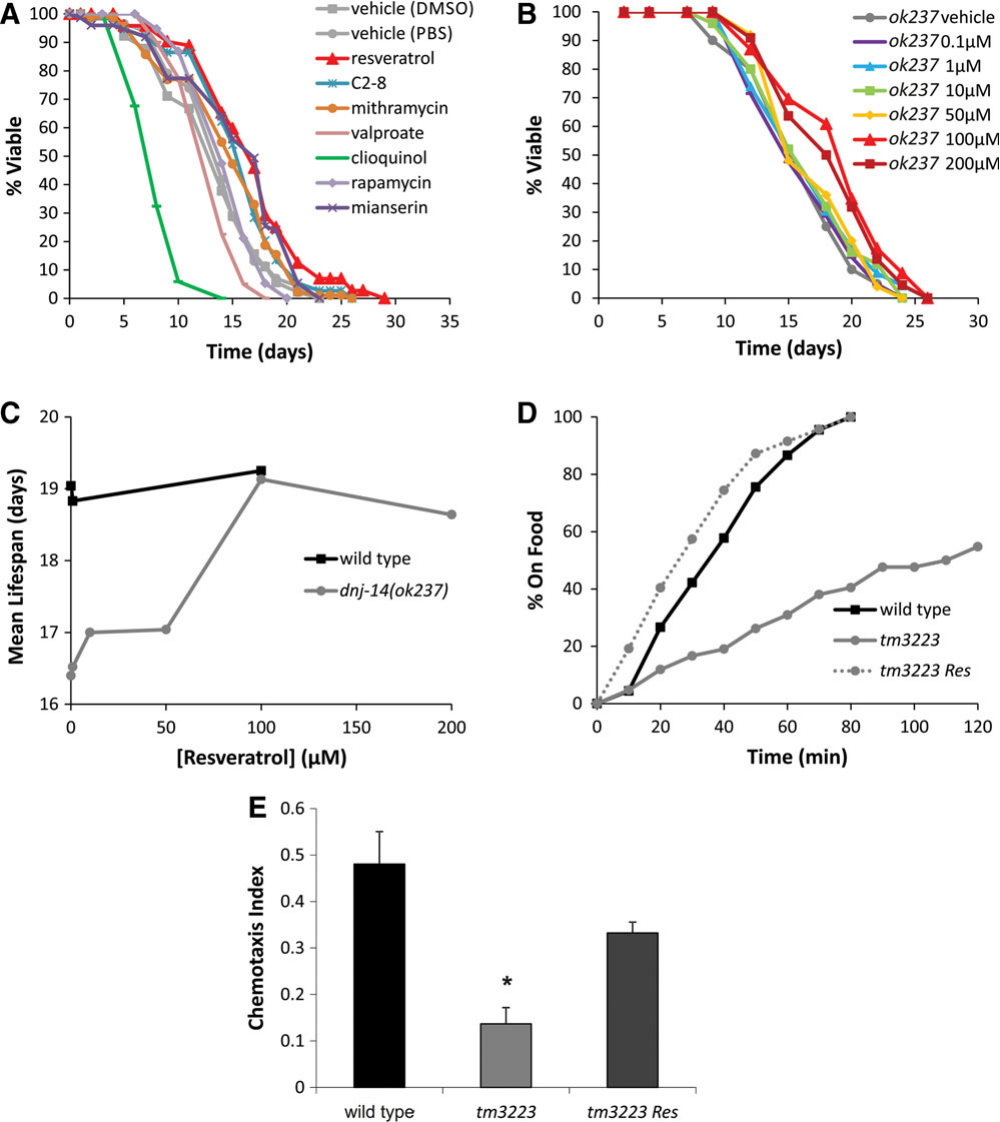
Resveratrol rescues *dnj-14* mutant phenotypes. (**A**) Drug screen for compounds able to increase the short lifespan of *dnj-14* mutants identifies resveratrol. *dnj-14*(*ok237*) worms were grown from L4 stage on NGM plates containing the indicated compounds or appropriate vehicle controls. Data shown are pooled from two independent lifespan assays (*n* = 67 to 115 worms used per condition). (**B** and **C**) Concentration-dependency of resveratrol. *dnj-14*(*ok237*) worms were grown from L4 stage on NGM plates containing the indicated concentrations of resveratrol. Data shown are derived from a single lifespan assay using 21–25 worms for each drug concentration and are displayed as raw survival curves (B) and as mean lifespans (C). (**D**) Resveratrol rescues the *dnj-14* food sensing defect. The time taken for 6-day-old N2 and *dnj-14*(*tm3223*) worms grown in the absence or presence of 100 µm resveratrol to move to a bacterial food source was measured. Data shown are pooled from two independent assays (*n* = 42–47 worms per strain per condition). (**E**) Resveratrol rescues the *dnj-14* chemotaxis defect. The proportion of wild-type and *dnj-14*(*tm3223*) worms that had moved to the attractant, isoamyl alcohol, within 90 min was measured in 2-day-old animals grown in the absence or presence of 100 µm resveratrol. Data shown are pooled from three independent assays (*n* = 85–102 worms per strain per condition; *P < 0.05).

We reasoned that the *dnj-14* model could be used to identify compounds with therapeutic potential for ANCL and possibly other neurodegenerative diseases. To test this idea, we screened a set of compounds for their ability to increase the short lifespan of *dnj-14* mutants. The compounds were chosen based on their reported beneficial effects on various different neurodegenerative disease models or on lifespan in wild-type worms (Supplementary Material, Table S1). Lifespan assays of wild-type and *dnj-14*(*ok237*) worms were performed by transferring untreated age-synchronized L4-stage animals onto NGM plates containing the appropriate compounds or vehicle controls (Fig. 4A). This first screen identified several compounds that affected lifespan in *dnj-14* mutants. Upon re-testing of these compounds in repeat experiments, only resveratrol was found to reproducibly and significantly extend lifespan in *dnj-14*(*ok237*) worms. This effect was concentration-dependent, with 100 µm resveratrol producing maximal lifespan extension (Fig. 4B and C). In contrast, resveratrol did not significantly increase lifespan in N2 control worms (Fig. 4C), suggesting a specific rescue of the defects caused by loss of DNJ-14. Resveratrol produced a partial rescue of the neurodegeneration phenotype (Supplementary Material, Fig. S2); and was also able to significantly ameliorate the food race and chemotaxis defects of *dnj-14* mutants (Fig. 4D and E).

### Resveratrol activity is mimicked by phosphodiesterase inhibition and independent of SIR-2.1

**Figure 5.**
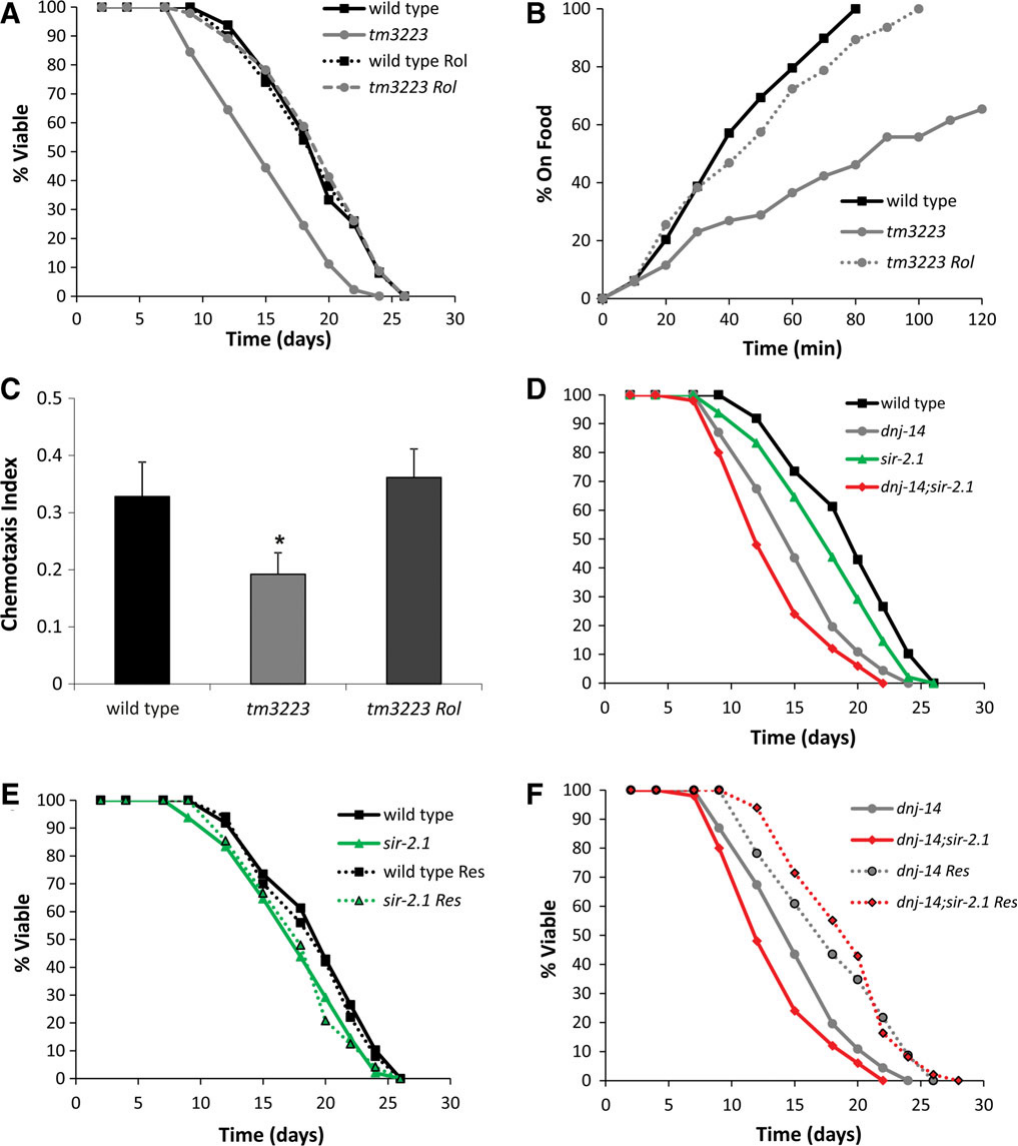
Resveratrol activity is mimicked by PDE inhibition and is independent of SIR-2.1. (**A**) Rolipram, a cAMP PDE inhibitor, rescues the short lifespan of *dnj-14* mutants. N2 or *dnj-14*(*tm3223*) worms were grown from L4 stage on NGM plates containing 100 µm rolipram or vehicle control. Data shown are pooled from two independent lifespan assays (*n* = 45–50 worms per condition). (**B**) Rolipram rescues the *dnj-14* food sensing defect. The time taken for 6-day-old N2 and *dnj-14*(*tm3223*) worms grown on NGM plates in the absence or presence of 100 µm rolipram to move to a bacterial food source was measured. Data shown are pooled from two independent assays (*n* = 47–52 worms per condition). (**C**) Rolipram rescues the *dnj-14* chemotaxis defect. The proportion of wild-type and *dnj-14*(*tm3223*) worms that had moved to the attractant, isoamyl alcohol, within 90 min was measured in 2–6-day-old animals grown in the absence or presence of 100 µm rolipram. Data shown are pooled from five independent assays (*n* = 151–160 worms per condition; *P < 0.05). (**D**–**F**) Effect of resveratrol on lifespan of *dnj-14* and *sir-2.1* mutants. Worms of the indicated genotypes were grown from L4 stage in the absence (D) or presence (E and F) of 100 µm resveratrol. Data shown are pooled from two independent lifespan assays comparing synchronized single and double mutant animals using 46–50 worms for each strain/condition.

The mechanism of action of resveratrol is unclear and controversial. Recent work has suggested that it acts as a cAMP phosphodiesterase (PDE) inhibitor (31), although this has not yet been confirmed by other studies. Consistent with this idea, we found that the PDE inhibitor, rolipram, mimicked the ability of resveratrol to rescue the short lifespan and sensory defects of *dnj-14* mutants (Fig. 5A–C). To directly test if resveratrol and rolipram acted via increasing cAMP levels, we performed cAMP assays on lysates from worms treated with these drugs for up to 2 h. Unfortunately, we did not even see an effect of the positive control rolipram (Supplementary Material, Fig. S6), so it was not possible to draw any conclusions from these experiments about the role of cAMP. Therefore, it remains possible that resveratrol and rolipram work through independent mechanisms to rescue the *dnj-14 m*utant phenotypes. Resveratrol is thought to activate (directly or indirectly) the Sirtuin class of NAD^+^-dependent histone deacetylases. Indeed, the effects of resveratrol in several *C. elegans* neurodegeneration models have been shown to be dependent on the Sirtuin, *sir-2.1* (32–34). We therefore constructed a double mutant *dnj-14;sir-2.1* strain to determine if this was also the case for our ANCL model. The *sir-2.1* mutation caused a small reduction in lifespan in both the wild-type and *dnj-14* backgrounds (Fig. 5D), consistent with previous findings. Resveratrol had no significant effect on the lifespan of the N2 or *sir-2.1* strains (Fig. 5E). Strikingly, the ability of resveratrol to increase longevity in the single *dnj-14* strain was fully maintained in the double mutant *dnj-14;sir-2.1* strain (Fig. 5F). This therefore suggests that resveratrol's protective effects are mediated via a *sir-2.1*-independent mechanism that is distinct from previously published worm neurodegeneration models.

## DISCUSSION

In this study, we report the phenotypes of *C. elegans* containing mutations in *dnj-14*, the orthologue of the CSP-encoding *DNAJC5* gene that is mutated in ANCL. *dnj-14* mutants are almost indistinguishable from wild-type worms when young, but have a reduced lifespan and develop profound sensory neuron defects with increasing age. The latter effect shows selectivity for sensory neuron classes, as mechanosensory neurons are unaffected at age points when chemosensation is severely impaired, thus ruling out general sickness effects. This impaired chemosensory function is already severe by Day 5, whereas visible abnormalities in sensory neurons are only evident from ∼9 days of age; hence functional synaptic deficits precede neurodegeneration in our *dnj-14 m*odel, similar to other neurodegenerative disease models (23). These phenotypes have some similarities to ANCL, which is characterized by adult onset (around age 30), short lifespan and selectivity in the types of neurons affected—for example, the visual system tends to be unaffected in ANCL, unlike other NCLs that are generally associated with blindness (6,7). The worm *dnj-14* phenotype is also reminiscent of *dnajc5* knockout mice, which are indistinguishable from wild-type littermates for the first few weeks, but have a short lifespan associated with progressive sensorimotor defects and neurodegeneration (16). These mice also show selective vulnerability of certain classes of neurons, in that hippocampal GABAergic neurons degenerate at time-points where glutamatergic transmission is unaffected (35). In contrast, *Drosophila csp* null mutations cause 95% embryonic lethality, with the few flies surviving to adulthood being extremely sick and short lived (11,12). It has been suggested (36) (but disputed (16,37)) that the relatively mild phenotype of mouse *dnajc5* mutants may be due to compensatory expression of CSPβ, which is highly homologous to CSPα but not present in *Drosophila*. However, as the worm genome contains only one CSP homologue, it appears more likely that the initially mild but progressive, age-dependent phenotypes shared by humans, mice and nematodes reflect the conserved functions of CSPα. The extreme severity of fly *csp* mutants coupled with the unfeasibility of performing high throughput screens in mice suggests that the worm *dnj-14* model may be an attractive new platform for chemical genetic screens for neuroprotective factors.

The focussed screen performed here revealed a potent protective effect of resveratrol on *dnj-14* mutant phenotypes. Resveratrol is a naturally occurring polyphenolic compound that has cardioprotective, anti-inflammatory, anti-tumour and neuroprotective properties (38). Although various mechanisms of action have been proposed, most attention has focussed on resveratrol's ability to activate the Sirtuin class of NAD^+^-dependent histone deacetylases, notably SIRT1. Indeed, various studies have shown that resveratrol action is dependent on SIRT1 in mammals and its homologue SIR-2.1 in *C. elegans* (32–34,39,40). However, whether resveratrol is a direct allosteric activator of Sirtuins (40) or instead affects Sirtuins indirectly via competitive inhibition of cAMP PDEs (31) remains controversial. Our finding that resveratrol's rescue of *dnj-14* phenotypes is undiminished by *sir–2.1* mutation demonstrates that SIR-2.1 activation is not essential for all resveratrol effects, in contrast with some (32–34) but not all (41) *C. elegans* studies.

The *dnj-14* model could shed light on the mechanism of action of resveratrol and, indeed, our observation that rolipram mimics the effect of resveratrol in rescuing *dnj-14* mutants is consistent with the recent discovery that resveratrol is an inhibitor of cAMP PDEs (31). Rolipram, like resveratrol, has cardioprotective and anti-inflammatory properties and has been shown to be neuroprotective in animal models of Alzheimer's disease and axonal degeneration (42,43). Interestingly, acute application of forskolin, which increases cAMP levels by activation of adenylate cyclase rather than PDE inhibition, can ameliorate the neuromuscular transmission defects in mouse *dnajc5* null mutants (44). Given that misfolding of SNAP-25 and hence reduced SNARE complex formation are thought to be the downstream consequence of impaired CSP function (18,19), it may be that resveratrol and rolipram compensate for this by activating alternative pathways resulting in improved SNARE complex formation, as seen with synuclein overexpression and proteasome inhibition (5,17). Indeed, phosphorylation of SNAP-25 and other synaptic exocytosis proteins by cAMP-dependent protein kinase is known to increase the pool of releasable synaptic vesicles, resulting in greater SNARE-dependent neurotransmission (45). However, as attempts to measure cAMP increases by resveratrol and rolipram in worms were unsuccessful, we cannot rule out the possibility that rescue of the *dnj-14 m*utant phenotypes by these compounds occurs via alternative mechanisms that are independent of cAMP. Nevertheless, the observation that treatment with resveratrol can mitigate the loss of CSP function in a Sirtuin-independent manner suggests that this may be a therapeutic strategy for patients with ANCL and potentially other neurodegenerative diseases.

## MATERIALS AND METHODS

### Materials

*C. elegans* strains RM2754 dnj-14(ok237), GR1373 *eri-1*(*mg366*) and VC199 *sir-2.1(ok434)* were obtained from the *Caenorhabditis* Genetics Center (CGC; University of Minnesota, Twin Cities, MN, USA). The TM3223 *dnj-14(tm3223)* strain was obtained from the National Bioresource Project for the Experimental Animal “Nematode *C. elegans*” based on the lab of Dr Shohei Mitani (Tokyo Women's Medical University, Tokyo, Japan). The *dnj-14* RNAi plasmid was obtained from Gene Service (Cambridge, UK). The pRAB100 and pPD117.01 plasmids were gifts from Drs Michael Nonet (Washington University, USA) and Andrew Fire (Stanford University, USA), respectively. All other materials were from Sigma (Poole, UK) unless stated otherwise.

### Strain construction and verification

Transgenic strains were generated by germline injection of expression constructs (5–20 ng/μl) as previously described (46). To visualize neurons, strains were injected with pRAB100, which carries GFP under the control of the pan-neuronal *rab-3* promoter (47). For transgenic rescue experiments, a construct comprising the genomic coding region of *dnj-14* plus 560 bp of upstream flanking sequence was inserted into a pPD117.01 plasmid with the GFP-coding region removed. Outcrossing was performed by mating with the wild-type N2 strain. The *ok237* strain was outcrossed six times and the *tm3223* strain outcrossed five times. The double mutant AMG74 *dnj-*14(tm3223);sir-2.1(ok434) strain was created by mating the outcrossed TM3223 *dnj-14(tm3223)* strain with the VC199 *sir-2.1(ok434)* strain and selecting for stable homozygous double mutant progeny. Strain genotypes were verified by single worm PCR analysis using the following primers:

**Table:** 

No.	Primer name	Sequence 5′–3′
1	dnj-14 del rev	TGCTCTCAACAGACCCATAC
2	dnj-14 del mid fwd	ATTCGCACGATCCGAAAAAG
3	dnj-14 del full fwd	CTGCATAGGGCACTCCTAGAAAT
4	tm3223 del full fwd	GCTTGTCTTACCTTATGTCGTCG
5	sir-2.1 del rev	CAGATAGTTCATACTGAAAATCT
6	sir-2.1 del fwd	AATCCCAATTGAACTCGCTG

### Nematode culture

*C. elegans* were grown under standard conditions on nematode growth media (NGM; 2% (w/v) agar, 0.3% (w/v) NaCl, 0.25% (w/v) peptone, 1 mm CaCl_2_, 5 μg ml^−1^ cholesterol, 25 mm KH_2_PO_4_, 1 mm MgSO_4_) agar plates. *Escherichia coli* OP50 was used as a food source; except for RNAi experiments, where the *E. coli* strain HT115 carrying the pG-L4440 vector was used. The wild-type reference strain was Bristol N2 except for RNAi experiments, where the *eri-1* hypersensitive strain was used. All strains were cultured and assays performed at 20°C.

### RNAi experiments

NGM plates containing 25 μg/ml carbenicillin and 1 mm IPTG were seeded with transformed HT115 bacteria and allowed to induce overnight. L3–L4 stage worms were transferred to each plate and once the next generation of worms had reached young adult stage, three adults were moved onto individual replica plates, allowed to lay eggs and removed the following day. These progeny were used in the assays once they had reached adulthood. To determine the extent of RNAi-mediated knockdown of *dnj-14* expression, worms were harvested after 3 days on RNAi or control plates. Total RNA was extracted using TRIzol (Invitrogen, UK) and equal amounts of RNA (1 μg) were incubated with reverse transcriptase and random hexamers (Promega, UK) to generate first-strand cDNA. This cDNA was amplified by PCR using primers for *dnj-14*, or for the β-actin gene, *act-1*, as control.

### Locomotion assays

Locomotion was measured in solution and quantified as thrashes per minute (one thrash defined as one complete sinusoidal movement from maximum to minimum amplitude and back again), as previously described (46). Single hermaphrodites were removed from NGM plates and placed in a Petri dish containing 200 μl freshly made Dent's solution (140 mm NaCl, 6 mm KCl, 1 mm CaCl_2_, 1 mm MgCl_2_, 5 mm HEPES, pH 7.4 with bovine serum albumin at 0.1 mg/ml). Assessment of locomotion was performed 10 min following immersion in solution. At least 10 animals were recorded per strain tested per experiment.

### Aldicarb assays

Acute sensitivity to aldicarb was assessed by measuring time to onset of paralysis following exposure to the drug, as previously described (26,48,49). For each experiment 20–30 animals were placed onto the centre of an unseeded NGM plate containing 1 mm aldicarb. To assess paralysis worms were lightly mechanically stimulated every 5–10 min following drug exposure. Worms were scored as paralyzed when they failed to respond to stimulation and pharyngeal pumping had ceased.

### Lifespan assays

Prior to lifespan analysis, worms were synchronized either by bleaching or by timed egg laying. Worms were then cleaned twice by washing in M9 buffer (5 g l^−1^ NaCl, 3 g l^−1^ KH_2_PO_4_, 6 g l^−1^ Na_2_HPO_4_, 1 mm MgSO_4_) for 10 min each before picking onto lifespan plates. Worms were picked onto fresh plates every alternate day to remove progeny, and were then checked for survival until all worms on the plates were dead. Worms were scored as dead when they failed to respond to mechanical stimulation from a tungsten wire or when pharyngeal pumping had ceased. At least 25 worms were used in each lifespan assay and all lifespan assays were repeated at least twice.

### Food race and chemotaxis assays

Food race assays were based on a previously described method (50). Briefly, NGM plates were poured three days prior to use on 60 mm petri dishes. They were seeded with 30 μl OP50 10 mm from the edge of the plate. Twenty to 30 age-synchronized worms were then washed twice in M9 buffer and placed 10 mm from the edge opposite to the food. The number of worms on the food was recorded, and those animals removed, every 10 min for two hours. Chemotaxis assays were based on a previously described method (51). Briefly, plates were made three days prior to use on 100 mm petri dishes containing 2% (w/v) agar, 5 mm KH_2_PO_4_, 1 mm CaCl_2_, 1 mm MgSO_4_. Worms were washed in M9 buffer and placed on the centre of the plate. One microlitre of the attractant and the diluent (ethanol) were pipetted on opposite edges of the plate. The number of worms on either side of the plate was counted after 90 min. The Chemotaxis Index was calculated as CI = (number of worms at attractant − number of worms at control)/total number of worms. A value of CI = 0.11 was considered as baseline for false positives, as previously established (51).

### Microscopy

Worms were age synchronized and divided into age groups of young (<7 days), middle-aged (9–12 days) and old (>12 days). Animals were immobilized in a solution of 20:20 PEG:glycerol in phosphate buffered saline (PBS) and imaged on a Nikon Eclipse Ti S fluorescence microscope with a ×20 objective and processed using the software NIS Elements. The neurodegeneration phenotype was classified based on altered neuronal GFP fluorescence as (a) loss of neuronal cell bodies, a reduction in the number of visible neurites, or the presence of contorted neuronal processes in the head of the worms, or (b) appearance of large fluorescent punctae within the dorsal nerve cord. The presence or absence of these phenotypes was used as a scoring system to quantify the phenotype.

### Drug treatment

Individual compounds were dissolved in PBS, dimethyl sulfoxide (DMSO) or ethanol and then added to molten NGM agar before pouring into petri dishes. Control NGM plates contained the appropriate dilution of vehicle. For the lifespan screen, all plates contained FUDR to prevent progeny overgrowth. Plates were allowed to dry for 1–2 days before seeding with OP50 bacteria. Developmentally synchronized worms prepared as described above were transferred onto the drug plates at L4 stage. For lifespan and neurodegeneration assays, worms were imaged for GFP fluorescence and assessed for viability every 2 days, and were transferred to fresh chemical plates as required. For food race and chemotaxis assays, resveratrol and rolipram were added to all media, including M9 washing buffers and assay plates.

### Cyclic AMP assay

This was based on a previously published method (52) using a commercial cAMP ELISA kit (GE Healthcare). Worms were synchronized by bleaching and grown on NGM plates seeded with JB1669 adenylyl cyclase deficient bacteria (Genotype: JB1669 F-glnV44(AS) recA1 endA1 gyrA96(NalR) thi1 hsdR17 spoT1 rfbD1 cyaA). Approximately fifteen 60 mm plates of Day 2 worms were washed off into M9 solution. The worms were allowed to settle, supernatant removed and washed again in 10 ml M9 buffer. This was repeated a further two times. After removing the supernatant the worms were made up to a final volume of 3 ml and split into three eppendorfs (1 ml per tube). These worms were treated with vehicle control (ethanol), resveratrol or rolipram for up to 2 h at room temperature on a rocker. The worms were then pelleted and the supernatant reduced to 100 µl. To this, 350 µl of M9 buffer was added plus 50 µl of Lysis Reagent 1A from the cAMP assay kit. Worms were vortexed for 30 s, sonicated three times for 9 s, and vortexed again. The samples were centrifuged to pellet any debris and the supernatant removed for use in the enzyme-linked immunosorbent assay (ELISA) and for protein estimation. Samples were both used straight or diluted 1 in 2. Cyclic AMP concentrations were determined by ELISA following the manufacturer's instructions and normalized for protein concentration.

### Mechanosensation and pharyngeal pumping assays

Prior to assays, worms were synchronized and grown on NGM plates seeded with OP50 bacteria until 6-days old. Mechanosensation assays were based on a previously described method (53). A male eyelash was plucked and the root end of this was glued to a toothpick, leaving the tapered end free. Before assaying, this was sterilized by dipping in 70% ethanol and subsequently air-dried. The worms were then transferred on to an unseeded 35 mm plate and left for 5min to acclimatize. Mechanosensation was assessed by gently touching the eyelash to the side of the worm's head at a perpendicular angle. Each worm was assayed 10 times and the number of times the worm either stopped or reversed its direction of movement was recorded. For pharyngeal pumping assays, worms feeding on OP50 bacteria were observed under ×80 magnification using a stereomicroscope. The number of contraction/relaxation cycles or pumps per thirty seconds was then counted for each worm.

### Statistical analysis

Statistical significance was assessed using Students' *t*-tests for behavioural assays. Log-rank tests were employed for aldicarb and lifespan assays, using the OASIS online service (54).

## SUPPLEMENTARY MATERIAL

Supplementary Material is available at *HMG* online.

## FUNDING

This work was supported by grants from the BBSRC (A.M., R.D.B. and J.W.B.), Research into Ageing (A.M.) and the Wellcome Trust (J.W.B., R.D.B. and A.M.). S.S.K. was supported by the Research into Ageing Fund, a fund set up and managed by Age UK. X.C. is supported by a BBSRC PhD studentship. R.C.J. and M.J.E. were supported by PhD studentships from the Wellcome Trust. Strains used in this work were provided by the *Caenorhabditis* Genetics Center, which is funded by the National Institutes of Health National Center for Research Resources (NCRR); and by the National Bio-Resource Project of *C. elegans*. Funding to pay the Open Access publication charges for this article was provided by the University of Liverpool Library.

## Supplementary Material

Supplementary Data
