# Supplementary material for: Caenorhabditis elegans dnj-14, the orthologue of the DNAJC5 gene mutated in adult onset neuronal ceroid lipofuscinosis, provides a new platform for neuroprotective drug screening and identifies a SIR-2.1-independent action of resveratrol
Source: Hum Mol Genet. 2014 Jun 19;23(22):5916–27. doi: 10.1093/hmg/ddu316 (PMC4204773; doi:10.1093/hmg/ddu316)
Supplement: Supplementary Data [file supp_23_22_5916__index.html]

Caenorhabditis elegans dnj-14, the orthologue of the DNAJC5 gene mutated in adult onset neuronal ceroid lipofuscinosis, provides a new platform for neuroprotective drug screening and identifies a SIR-2.1-independent action of resveratrol — Caenorhabditis elegans dnj-14, the orthologue of the DNAJC5 gene mutated in adult onset neuronal ceroid lipofuscinosis, provides a new platform for neuroprotective drug screening and identifies a SIR-2.1-independent action of resveratrol — Supplementary Data 

# *Caenorhabditis elegans dnj-14*, the orthologue of the *DNAJC5* gene mutated in adult onset neuronal ceroid lipofuscinosis, provides a new platform for neuroprotective drug screening and identifies a SIR-2.1-independent action of resveratrol

## Supplementary Data

Supplementary Data

**Files in this Data Supplement:**

- Supplementary Data - Pdf file
- Supplementary Video - wmv file
