# Supplementary material for: Caenorhabditis elegans dnj-14, the orthologue of the DNAJC5 gene mutated in adult onset neuronal ceroid lipofuscinosis, provides a new platform for neuroprotective drug screening and identifies a SIR-2.1-independent action of resveratrol
Source: Hum Mol Genet. 2014 Jun 19;23(22):5916–27. doi: 10.1093/hmg/ddu316 (PMC4204773; doi:10.1093/hmg/ddu316)
Supplement: Supplementary Data [file supp_ddu316_ddu316supp_data.pdf]

Supplementary Information

Supplementary Table

Compound	Suggested Mechanisms	Assay	Reference
**Resveratrol**	Sirtuin activator, PDE inhibitor, antioxidant	Worm PolyQ neuroprotection<br>Mouse amyloid plaque reduction	(1)<br>(2)
**Mithramycin**	DNA binding agent	Worm PolyQ neuroprotection<br>Mouse HD survival	(3)<br>(4)
**Clioquinol**	Metal chelator and<br>CLK-1 inhibitor	Worm *clk-1* partial mimic<br>Mouse Alzheimer cognition	(5)<br>(6)
**Valproic acid**	Anticonvulsant<br>(HDAC inhibitor)	Worm lifespan increase<br>Fly PolyQ neuroprotection	(7)<br>(8)
**Mianserin**	Antidepressant<br>(5-HT2 blocker)	Worm lifespan increase	(9)
**C2–8**	unknown	Fly PolyQ neuroprotection<br>Mouse HD survival	(10)<br>(11)
**Rapamycin**	mTOR inhibitor	Cell synuclein/PolyQ<br>Mouse AD cognition	(12)<br>(13)

Table S1: Compounds used in *dnj-14* lifespan screen.

Figure legends

FIG. S1: *dnj-14* gene structure, mutations and homology to human *dnajc5*.

(A) Sequence alignment of the predicted products of the human *DNAJC5* and *C. elegans dnj-14a* genes (CSPα and DNJ-14 proteins, respectively).

(B) Exon structure of the *dnj-14* gene and location of the *ok237* and *tm3223* alleles.

(C) Confirmation of the *ok327* deletion. Genomic DNA from wild type N2 and *dnj-14(ok237)* worms was amplified using primers flanking the deletion (left panel) or with one flanking primer and a primer within exon 2 of *dnj-14* (right panel).

(D) Characterisation of the *tm3223* insertion/deletion. Genomic DNA from wild type N2 and *dnj-14(tm3223)* worms was amplified using primers flanking the mutation site.

FIG. S2: Quantification of neurodegeneration in *dnj-14(ok237)* mutants.

Wild type N2 and *dnj-14(ok237)* worms were synchronised and grown on NGM plates containing vehicle control (ethanol) or 100 μM resveratrol for at least 9 days. Animals were then immobilised for GFP imaging and scored for head neuron abnormalities ('neuron loss') based on loss of neuronal cell bodies, a reduction in the number of visible neurites, or the presence of contorted neuronal processes in the head of the worms; and the presence of large fluorescent punctae in the dorsal nerve cord ('punctae'). The number of worms analysed was 45 for N2, 35 for *dnj-14* and 20 for *dnj-14* Resveratrol.

FIG. S3: Visualisation of neurodegeneration in *dnj-14(ok237)* mutants.

Wild type N2 and *dnj-14(ok237)* worms were synchronised and grown on NGM plates for at least 9 days. Animals were then immobilised for GFP imaging. Typical images are shown for various animals. These illustrate the loss of neuronal cell bodies, reduction in the number of

visible neurites, or the presence of contorted neuronal processes in the head of the worms that was frequently seen in *dnj-14* mutants. In contrast, age-matched wild type N2 animals generally exhibited obvious neuronal cell bodies and had clearly labelled multiple neurites that extended straight to the end of the worm's head without twisting.

FIG. S4: Mechanosensation is unaffected in aged *dnj-14* mutants.

Wild type N2, *dnj-14(tm3223)* and *dnj-14(ok237)* worms were synchronised and grown on NGM plates until 6 days of age. After transfer to unseeded plates, mechanosensation was assessed by gently touching an eyelash to the side of the worm's head at a perpendicular angle. Each worm was assayed ten times and the number of times the worm either stopped or reversed its direction of movement was recorded. No significant differences between wild type and *dnj-14* mutants were seen (n=15 animals per strain).

FIG. S5: Pharyngeal pumping is unaffected in aged *dnj-14* mutants.

Wild type N2, *dnj-14(tm3223)* and *dnj-14(ok237)* worms were synchronised and grown on NGM plates until 6 days of age. After transfer to plates freshly seeded with OP50 bacteria, the number of contraction/relaxation cycles of the pharynx per thirty seconds was for each worm was recorded. No significant differences between wild type and *dnj-14* mutants were seen (n=15 animals per strain).

FIG. S6: Measurement of cAMP levels in worms treated with resveratrol and rolipram.

Wild type N2 worms were age-synchronised and grown on 60-mm NGM plates seeded with JB1669 adenylyl cyclase deficient bacteria. Approximately fifteen such plates of day 2 worms were washed in M9 buffer and then treated in M9 buffer containing vehicle control (ethanol), 100 µM resveratrol or 100 µM rolipram for 120 mins. The worms were then lysed,

centrifuged to pellet any debris, and the supernatant used immediately for assay. Endogenous cyclic AMP levels were measured by ELISA and normalised to total protein concentration. Data shown are mean + SEM (*n* = 4 biological replicates). No significant differences between treatments were seen.

MOVIE S1: Superficially normal locomotion in *dnj-14(ok237)* mutants

The movement of worms on NGM agar plates was recorded. Wild type N2 worms are shown first, followed by *dnj-14(ok237)* mutants.

Supplementary References

1 Parker, J.A., Arango, M., Abderrahmane, S., Lambert, E., Tourette, C., Catoire, H. and Neri, C. (2005) Resveratrol rescues mutant polyglutamine cytotoxicity in nematode and mammalian neurons. *Nat Genet*, **37**, 349-350.

2 Karuppagounder, S.S., Pinto, J.T., Xu, H., Chen, H.L., Beal, M.F. and Gibson, G.E. (2009) Dietary supplementation with resveratrol reduces plaque pathology in a transgenic model of Alzheimer's disease. *Neurochem Int*, **54**, 111-118.

3 Voisine, C., Varma, H., Walker, N., Bates, E.A., Stockwell, B.R. and Hart, A.C. (2007) Identification of potential therapeutic drugs for huntington's disease using Caenorhabditis elegans. *PLoS One*, **2**, e504.

4 Ferrante, R.J., Ryu, H., Kubilus, J.K., D'Mello, S., Sugars, K.L., Lee, J., Lu, P., Smith, K., Browne, S., Beal, M.F. *et al.* (2004) Chemotherapy for the brain: the antitumor antibiotic mithramycin prolongs survival in a mouse model of Huntington's disease. *J Neurosci*, **24**, 10335-10342.

5 Wang, Y., Branicky, R., Stepanyan, Z., Carroll, M., Guimond, M.P., Hihi, A., Hayes, S., McBride, K. and Hekimi, S. (2009) The anti-neurodegeneration drug clioquinol inhibits the aging-associated protein CLK-1. *J Biol Chem*, **284**, 314-323.

6 Adlard, P.A., Cherny, R.A., Finkelstein, D.I., Gautier, E., Robb, E., Cortes, M., Volitakis, I., Liu, X., Smith, J.P., Perez, K. *et al.* (2008) Rapid restoration of cognition in Alzheimer's transgenic mice with 8-hydroxy quinoline analogs is associated with decreased interstitial Abeta. *Neuron*, **59**, 43-55.

7 Evason, K., Collins, J.J., Huang, C., Hughes, S. and Kornfeld, K. (2008) Valproic acid extends Caenorhabditis elegans lifespan. *Aging Cell*, **7**, 305-317.

8 Yi, J., Zhang, L., Tang, B., Han, W., Zhou, Y., Chen, Z., Jia, D. and Jiang, H. (2013) Sodium valproate alleviates neurodegeneration in SCA3/MJD via suppressing apoptosis and rescuing the hypoacetylation levels of histone H3 and H4. *PLoS ONE*, **8**, e54792.

9 Petrascheck, M., Ye, X. and Buck, L.B. (2007) An antidepressant that extends lifespan in adult Caenorhabditis elegans. *Nature*, **450**, 553-556.

10 Zhang, X., Smith, D.L., Meriin, A.B., Engemann, S., Russel, D.E., Roark, M., Washington, S.L., Maxwell, M.M., Marsh, J.L., Thompson, L.M. *et al.* (2005) A potent small molecule inhibits polyglutamine aggregation in Huntington's disease neurons and suppresses neurodegeneration in vivo. *Proc Natl Acad Sci U S A*, **102**, 892-897.

11 Chopra, V., Fox, J.H., Lieberman, G., Dorsey, K., Matson, W., Waldmeier, P., Housman, D.E., Kazantsev, A., Young, A.B. and Hersch, S. (2007) A small-molecule therapeutic lead for Huntington's disease: preclinical pharmacology and efficacy of C2-8 in the R6/2 transgenic mouse. *Proc Natl Acad Sci U S A*, **104**, 16685-16689.

12 Sarkar, S., Perlstein, E.O., Imarisio, S., Pineau, S., Cordenier, A., Maglathlin, R.L., Webster, J.A., Lewis, T.A., O'Kane, C.J., Schreiber, S.L. *et al.* (2007) Small molecules enhance autophagy and reduce toxicity in Huntington's disease models. *Nat Chem Biol*, **3**, 331-338.

13 Spilman, P., Podlutskaya, N., Hart, M.J., Debnath, J., Gorostiza, O., Bredesen, D., Richardson, A., Strong, R. and Galvan, V. (2010) Inhibition of mTOR by rapamycin abolishes cognitive deficits and reduces amyloid-beta levels in a mouse model of Alzheimer's disease. *PLoS ONE*, **5**, e9979.

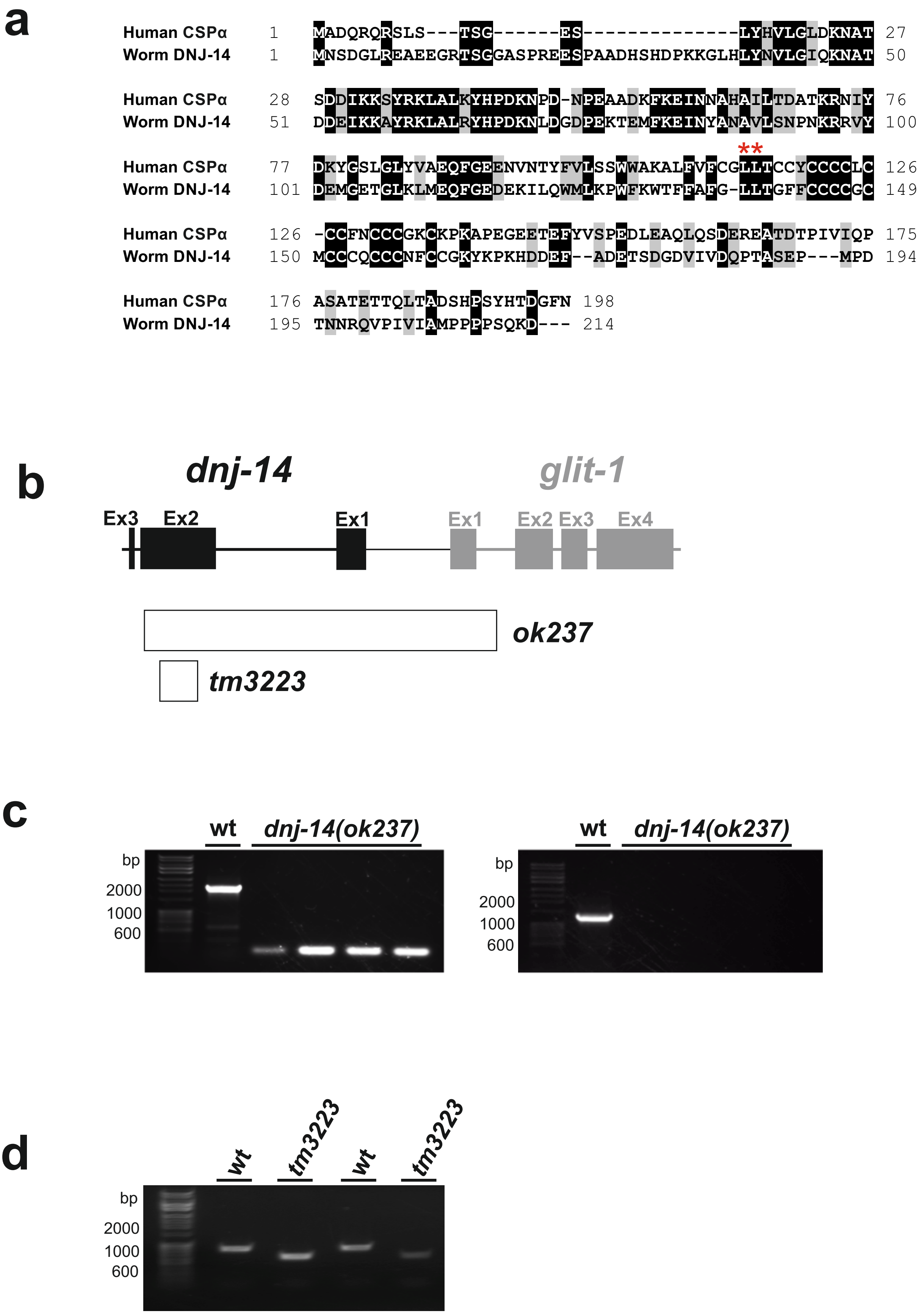

Fig S1 Kashyap et al

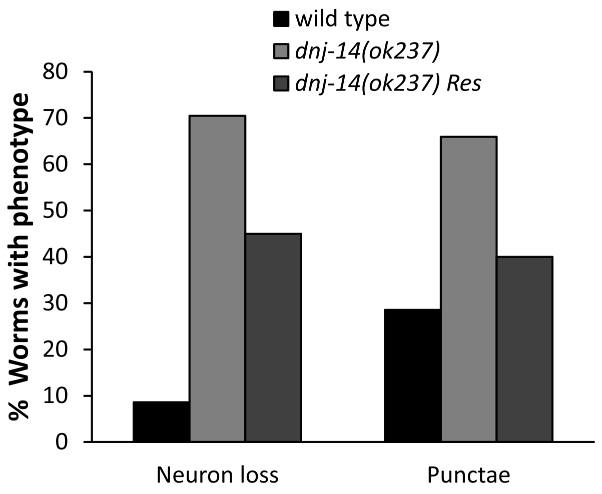

Fig S2 Kashyap et al

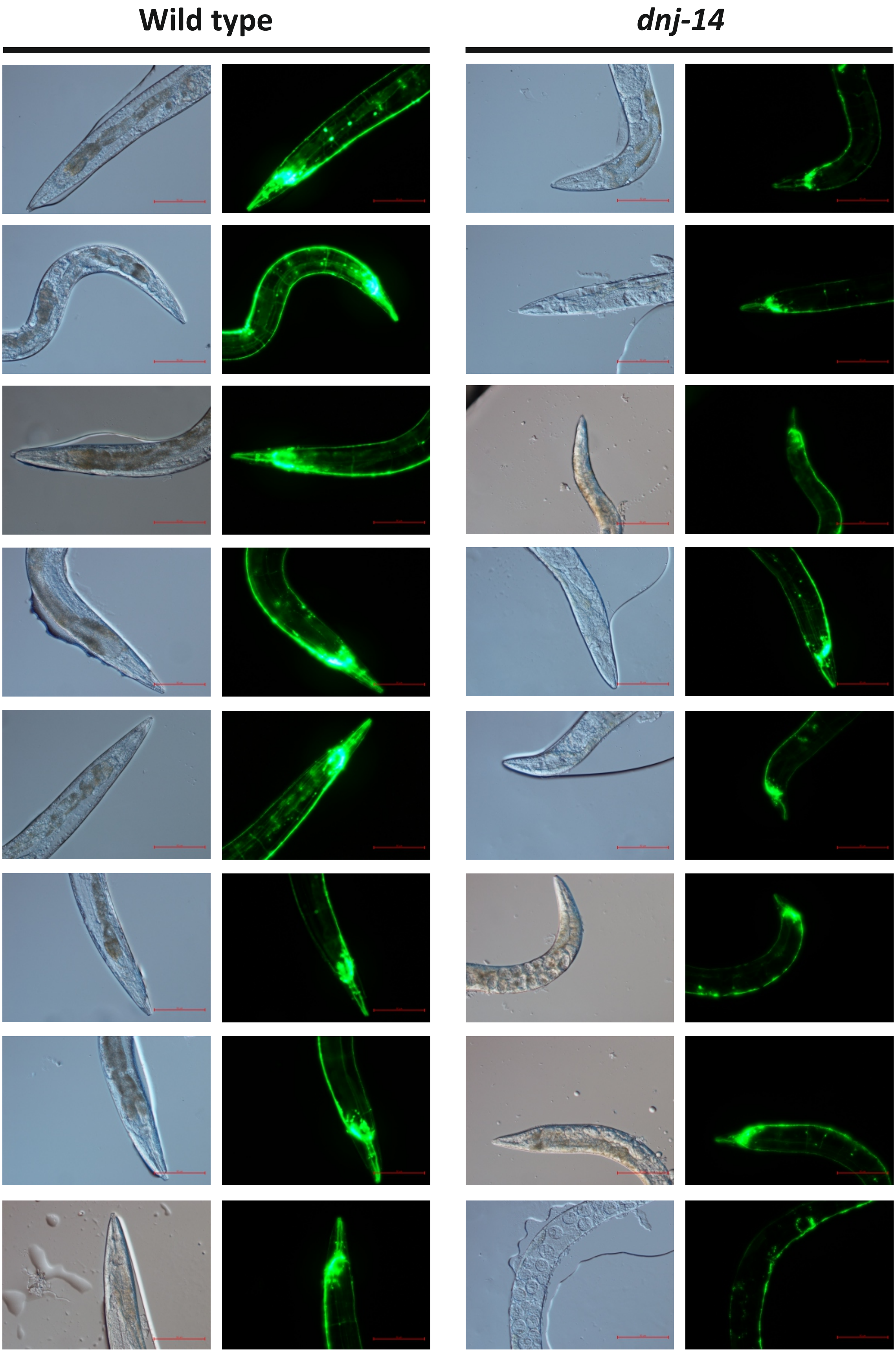

Fig S3 Kashyap et al

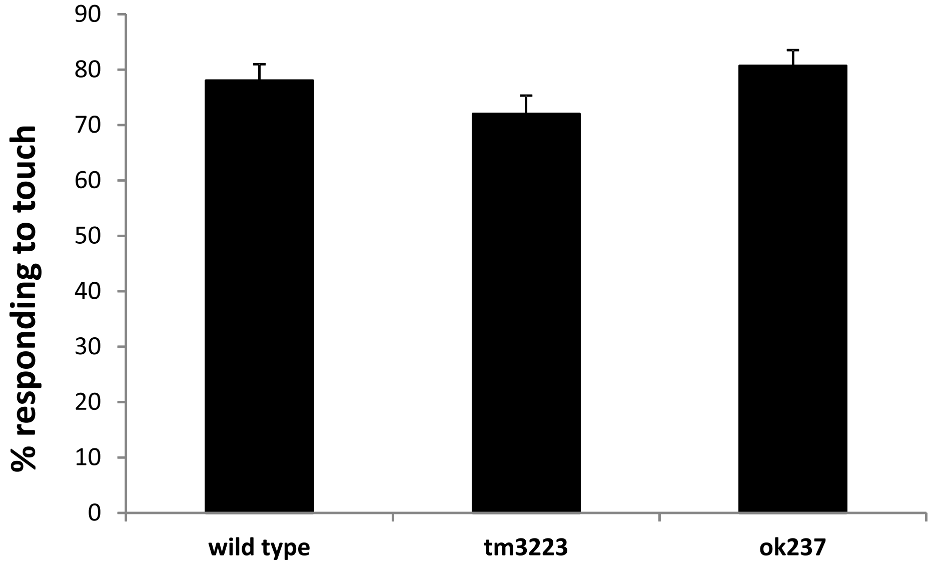

Fig S4 Kashyap et al

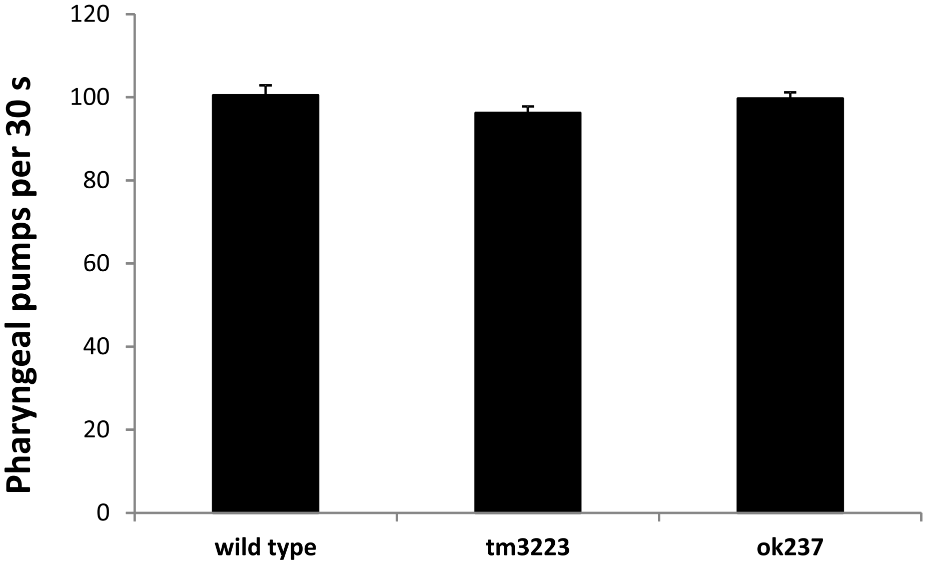

Fig S5 Kashyap et al

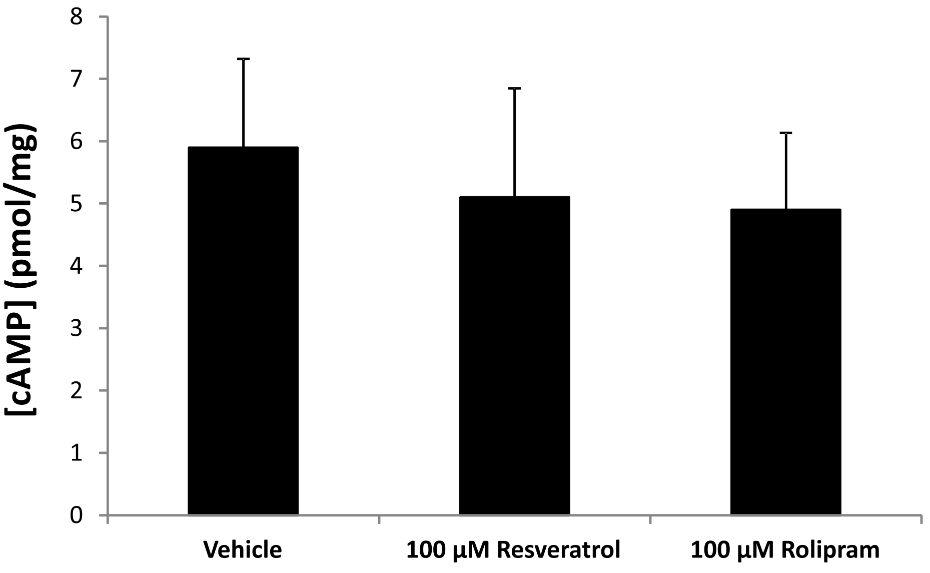

Fig S6 Kashyap et al
